# Trends in Prevalence of Hypertension in Brazil: A Systematic Review with Meta-Analysis

**DOI:** 10.1371/journal.pone.0048255

**Published:** 2012-10-31

**Authors:** Rafael V. Picon, Flávio D. Fuchs, Leila B. Moreira, Glaube Riegel, Sandra C. Fuchs

**Affiliations:** 1 Postgraduate Studies Program in Cardiology, School of Medicine, Universidade Federal do Rio Grande do Sul, Porto Alegre, Rio Grande do Sul, Brazil; 2 Hospital de Clinicas de Porto Alegre, Division of Cardiology, and the National Institute for Science and Technology for Health Technology Assessment (IATS/CNPq), Porto Alegre, Rio Grande do Sul, Brazil; Tehran University of Medical Sciences, Iran (Republic of Islamic)

## Abstract

**Background:**

The prevalence of hypertension in emerging nations was scarcely described to date. In Brazil, many population-based surveys evaluated the prevalence in cities throughout the country. However, there is no population-based nationwide study of prevalence of hypertension. In this study, we estimated the prevalence of hypertension for the country and analyzed the trends for the last three decades.

**Methods:**

Cross-sectional and cohort studies conducted from 1980 to 2010 were independently identified by two reviewers, without language restriction, in the PubMed, Embase, LILACS, and Scielo electronic databases. Unpublished studies were identified in the Brazilian electronic database of theses and in annals of Cardiology congresses and meetings. In total, 40 studies were selected, comprising 122,018 individuals.

**Results:**

Summary estimates of prevalence by the former WHO criteria (BP≥160/95 mmHg) in the 1980’s and 1990’s were 23.6% (95% CI 17.3–31.4%) and 19.6% (16.4–23.3%) respectively. The prevalence of hypertension by the JNC criteria (BP≥140/90 mmHg) in the 1980’s, 1990’s and 2000’s were 36.1% (95% CI 28.7–44.2%), 32.9% (29.9–36.0%), and 28.7% (26.2–31.4%), respectively (P<0.001). In the 2000’s, the pooled prevalence estimates of self-reported hypertension on telephone inquiries was 20.6% (19.0–22.4%), and of self-reported hypertension in home surveys was 25.2% (23.3–27.2%).

**Conclusions:**

The prevalence of hypertension in Brazil seems to have diminished 6% in the last three decades, but it still is approximately 30%. Nationwide surveys by self-reporting by telephone interviews underestimate the real prevalence. Rates of blood pressure control decreased in the same period, corresponding currently to only one quarter of individuals with hypertension.

## Introduction

Hypertension has become a growing public health concern, particularly in developing countries, with an estimated prevalence of 37.3%, in comparison with 22.9% in industrialized nations.^1^ Projections are that by the year of 2025, 75.0% (or 1.17 billion people) of the people with hypertension in the world will be living in emerging nations [1].

Although hypertension has been recognized as a major risk factor for cardiovascular morbidity and mortality worldwide, there are lacking nationwide prevalence data in most emerging countries [2], [3]. Such information is needed in order to determine the economic burden of hypertension, as well as to optimize health resources allocation toward improvement on its detection, treatment and control. In Brazil, many population-based surveys, representative of cities and of one state, have been done in the last three decades, but there is no estimate of prevalence for the whole country or of trends in this period. Hence, our study aimed to estimate the prevalence trends of hypertension in the adult Brazilian population through a systematic review with meta-analysis of population-based studies.

## Methods

### Study Designs and Eligibility Criteria

The eligibility criteria included population-based cross-sectional or cohort studies among participants aged 18 years or older, from 1980 to 2010. Studies with pregnant women were not included.

Studies with duplicate data were excluded. Population-based studies that addressed only specific socioeconomic strata (such as low-income individuals, or certain industry workers) were not considered representative of its geographical (city, State, or region) population and, therefore, deemed ineligible. Studies that assessed only secondary hypertension, or used samples originated from sources other than the general geographical population (i.e. not population-based) were also excluded.

### Information Sources

The search of the published literature was conducted in the electronic databases of PubMed, Embase, LILACS (Latin American and Caribbean Health Sciences Literature), and Scielo (Scientific Electronic Library Online) using MeSH terms and Entrees for PubMed e Embase, and DeCS (Health Sciences Descriptors) for the other two databases. Data that were not formally published were additionally searched in PhD theses and Master’s dissertations registered in the electronic database of the Coordination for the Improvement of Higher Education Personnel (CAPES), Ministry of Education, Brazil. Annals of national and regional scientific sessions of Cardiology in Brazil were searched to identify studies presented only in these meetings. Full-text version of all potentially relevant articles, theses, or dissertation were downloaded from electronic databases or requested directly to the authors via e-mail.

### Searching

All searches were carried out independently by two reviewers. Search strategies were tested with the key words “hypertension”, “prevalence”, “statistics”, and “Brazil”, using the Boolean operator “OR”, which retrieved tens of thousands of records. A second attempt was carried out in the same databases using the operator “AND”. The following search strategies were used on PubMed: (“Hypertension”[Majr] AND “Prevalence”) AND “Brazil” limited to all adults (≥19 years-old), and (“Hypertension/epidemiology”[Majr] OR “Hypertension/statistics and numerical data”[Majr]) AND “Brazil” limited to all adults (≥18 years-old). Only searches on PubMed and Embase were filtered for studies conducted in adults. No language restriction was applied. Independent manual search on reference lists of retrieved articles was also undertaken.

### Study Selection and Data Collection

The first screening was based on a double-screening of titles and abstracts. Results which met explicit exclusion criteria were excluded. In the second step, the remaining manuscripts were assessed for full-text reading. In case of disagreement among reviewers, a third reviewer assessed the study and a decision for inclusion was reached by consensus. Data were entered in a pre-tested Microsoft Office Excel™ spreadsheet that was designed based on the Strengthening the Reporting of Observational Studies in Epidemiology Statement (STROBE) checklist [4]. Items 4, 5, 6a, 7–10, 12c–e, 13a, 14b, 16a, and 17 of the STROBE checklist were taken into account for the development of the data extraction spreadsheet.

Hypertension prevalence was the main summary measure used in this systematic review, which was extracted from studies using different definitions, that comprised four diagnostic criteria: blood pressure (BP) ≥140/90 mmHg or use of BP lowering medication (BPLM) (hereafter the JNC criteria - according to the Fourth to Seventh Joint National Committee on Prevention, Detection, Evaluation, and Treatment of High Blood Pressure); BP≥160/95 mmHg or use of BPLM (henceforward former World Health Organization (WHO) criteria, employed in older studies); self-reported hypertension through home visits, and self-reported hypertension through telephone inquiries [6], [7]. Many studies with measured blood pressure presented estimates for the former WHO and JNC criteria, but older studies presented only for the former WHO criteria. Hypertension control rate was defined as the proportion of subjects with hypertension using BPLM and normal BP over the total number of subjects with hypertension on treatment.

### Assessment of Study Quality and Risk of Bias

All studies were assessed for selection and measurement biases as well as bias in the data analysis based on guidelines of the MOOSE checklist [5]. Selection biases were characterized by refusals to participate in the study of 20% or higher, description of a non-random sampling, the use of other than a random process for participants recruitment, and data collection made through telephone interviews, since it covers participants of higher socioeconomic level. Measurement biases were defined considering the type of device used for blood pressure measurement, the discard of the first measurement, except for studies that used self-reported hypertension or the report of lacking impact in the analysis. Bias in the analysis was considered possible if the design effect was not taken into account in calculating the prevalence of hypertension. All biases were dealt with sensitivity analyses, defined *a priori*, using the abovementioned factors stratified for diagnostic criteria and decade (e.g. oscillometric *vs.* all devices, according to JNC criteria in the 2000’s; studies adjusted to design effect *vs.* all studies, according to the former WHO criteria in the 1980’s; etc.). Also, a sensitivity analyses comparing the overall prevalence of hypertension, according to the JNC criteria in the 2000’s, versus the pooled prevalence without studies carried out exclusively in rural populations or studies that did not investigated elderly individuals was performed.

### Data Analysis

All point estimates of analyses and their 95% confidence interval (95% CI) were calculated using random effects models according to decade, sex (when possible), and hypertension definition. The random effects model, wherein the weight study is inversely proportional to the sum of the variation within and between study studies of variance (T2) allows one to study the variance is diluted in variance between studies. Therefore, study variance impact on study weight is considerably diminished, and so is the influence of individual studies weights to the model as a whole. Nevertheless, the analyses using fix effect models were also tested, resulting in identical point estimates, but with narrower confidence intervals (data not shown).

Subgroup analyses included overall prevalence of hypertension according to the JNC criteria by decade, analyses by macro-region and design effect correction in the 2000’s, and control rates from 1980 to 2010 by decade. Heterogeneity and consistency were evaluated through Cochran’s *Q* and the *I^2^* statistics, respectively. Analyses were performed using the second version of the Comprehensive Meta-Analysis™ software. Forest plots were constructed using an electronic spreadsheet developed by Neyeloff et al [8].

Chi-square (χ^2^) was used to assess difference in prevalence rates among two distinct decades. Chi for trend (χ for trend) was used to evaluate prevalence and control rate across the three decades. Meta-regression – regressing the year of data collection and local human development index (HDI) on the logit prevalence rate – was employed to assess the prevalence variation throughout the studied period, using the method of moments for the estimation of tau-squared (τ^2^, i.e. between-study variance).

The Institution Review Board, which is accredited by the US Office of Human Research Protections, approved the research protocol.

## Results

### Synthesis of Data

**Figure 1:**
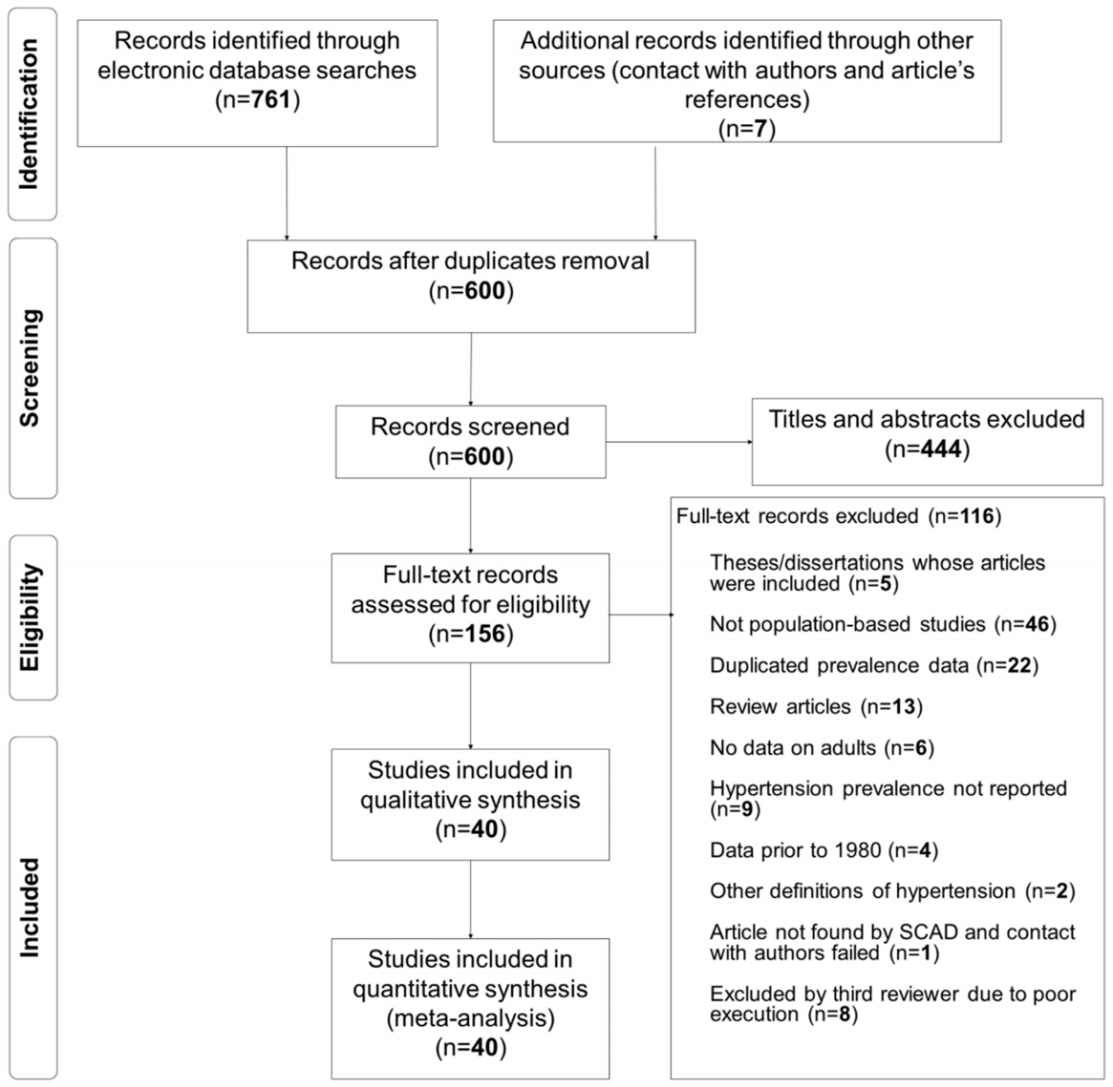
Flowchart of records retrieved, screened and included in the systematic review.

Through the searches, 761 articles were found in the electronic databases (51 being theses/dissertations published in the CAPES’s database), one study published by some of the authors was further analyzed to provide data, and other six articles retrieved by manual search – totalizing 600 initial records after removal of duplicates [9]. Manual search of the Annals of Cardiology meetings identified only studies already found in other sources. The first screening excluded 444 records and the second screening, another 108. By consensus with the third reviewer another eight studies were excluded, leaving 40 studies with 122018 individuals for the analysis. Agreement among reviewers for individual selection of studies was 78%, and after consensus meetings it reached 100%. Flowchart of studies selection is presented in Figure 1. The list of studies included and excluded in the meta-analysis, and the reasons for exclusion, are presented in Table S1.

Table 1 presents the overall characteristics of the 40 studies. Prevalence rates and their 95% CI by decade, diagnostic criteria, and method of assessment (measured or self-reported) are presented in Table 2. Summary estimates according to the former WHO criteria (BP≥160/95 mmHg or BPLM) in the 1980’s and 1990’s were 23.6% (95% CI 17.3–31.4%) and 19.6% (16.4–23.3%), respectively. In the 2000’s, the pooled prevalence estimates of self-reported hypertension on telephone inquiries was 20.6% (19.0–22.4%), and of self-reported hypertension in home surveys was 25.2% (23.3–27.2%).

**Table 1:** Source, year, sample size and methodological aspects of the 40 studies included in meta-analysis (in alphabetical order of first author).

First author	Source or database	Year of data collection	N	Age criterion for study entry (years)	City, State, or region	Devices used and hypertension assessment method	BP cut-off value for hypertension (SBP/DBP in mm Hg)
Costa VG	LILACS	1982	1200	18–80	Uberlândia	Device not described	160/95
de Lolio CA	PubMed & LILACS	1987	1199	15–74	Araraquara	Aneroid	140/90 & 160/95
Martins IS	Embase, LILACS, PubMed & Scielo	1987	1041	≥20	Cotia	Mercury & SRH	140/90 & 160/95
Ayres JE	PuMed	1988	1944	≥15	Piracicaba	Device not described	160/95
Fuchs FD	Embase, PubMed & LILACS	1989	1091	≥18	Porto Alegre	Aneroid, BPLM & SRH	140/90 & 160/95
Bloch KV	Embase, PubMed & LILACS	1991	1268	≥20	Rio de Janeiro city	Mercury & BPLM	160/95
Piccini RX	Embase, LILACS, PubMed &Scielo	1992	1657	20–69	Pelotas	Aneroid, BPLM & SRH	160/95
Trindade IS	Embase, LILACS, PubMed & Scielo	1995	206	≥18	Passo Fundo	Aneroid, BPLM & SRH	160/95
Fuchs SC^‡^	Embase, PubMed & LILACS	1996	1174	≥18	Porto Alegre	Aneroid & SRH	140/90
Barreto SM	Embase, LILACS, PubMed & Scielo	1997	820	18–59	Bambuí	Mercury & SRH	140/90
de Oliveira RZ	Embase & LILACS	1998	411	20–69	Cianorte	Mercury, BPLM & SRH	140/90 & 160/95
Freitas OC	Embase, LILACS, PubMed & Scielo	1998	688	≥18	Catanduva	Aneroid	140/90
da Costa JSD	LILACS & CAPES-TD	1999	1968	20–69	Pelotas	Aneroid & BPLM	160/95
Gus I	LILACS & Scielo	1999	1063	≥20	Rio Grande do Sul (state)	No description & SRH	140/90 & 160/95
Lessa I	Embase, LILACS, PubMed & Scielo	1999	1439	≥20	Salvador	Oscillometric & BPLM	140/90
First author	Source or database	Year of data collection	N	Age criterion for study entry (years)	City, State, or region	Devices used and hypertension assessment method	BP cut-off value for hypertension (SBP/DBP in mm Hg)
Mill JG	Article references & CAPES-TD	1999	1656	25–64	Vitória	Mercury & BPLM	140/90
Gimeno SGA	Embase	2000	201	≥20	Alto Xingu	Aneroid & Mercury	140/90
de Souza LJ	LILACS	2001	1039	≥18	Campos dos Goytacazes	Aneroid &, BPLM SRH	140/90
INCA**	Article reference	2002	17059	≥25	18 Capitals	SRH	Not applicable
Jardim PCBV	Embase, LILACS, PubMed & Scielo	2002	1739	≥18	Goiânia	Oscillometric & BPLM	140/90
Barbosa JB	Embase, LILACS, PubMed & Scielo	2003	835	≥18	São Luís	Aneroid & BPLM	140/90
Capilheira MF	LILACS	2003	3100	≥20	Pelotas	SRH	Not applicable
Cassanelli T	CAPES-TD	2003	1699	18–74	Cuiabá	Device not described & BPLM	140/90
de Souza JJG	LILACS & CAPES-TD	2003	1667	≥20	São Paulo city	SRH	Not applicable
Hartmann M	PubMed, LILACS, Scielo & CAPES-TD	2003	1020	20–60	São Leopoldo	Aneroid & BPLM	140/90
Matos AC	LILACS	2003	126	≥19	Cavunge	Aneroid & BPLM	140/90
Monteiro CA*	Article references	2003	2122	≥18	São Paulo city	SRH	Not applicable
Carvalhaes MABL*	LILACS	2004	1410	≥18	Botucatu	SRH	Not applicable
Cesarino CB	LILACS & Scielo	2004	1717	≥18	São José do Rio Preto	Device not described & BPLM	140/90
de Castro RA	Embase, LILACS, PubMed & Scielo	2004	285	≥18	Formiga	Oscillometric	140/90
First author	Source or database	Year of data collection	N	Age criterion for study entry (years)	City, State, or region	Devices used and hypertension assessment method	BP cut-off value for hypertension (SBP/DBP in mm Hg)
Borges HP*	Embase, LILACS & Scielo	2005	2352	≥18	Belém	SRH	Not applicable
Fuchs SC^†^	Article reference	2005	1007	≥18	Porto Alegre	Oscillometric, BPLM & SRH	140/90
Peixoto MRG*	LILACS	2005	2002	≥18	Goiânia	SRH	Not applicable
SOFT study	Directly from the author	2005	1858	18–90	Porto Alegre	Oscillometric & BPLM	140/90
Ferreira SRHG*	Pubmed	2006	54369	≥18	Brasília & state capitals	SRH	Not applicable
Nunes Filho JR	LILACS	2006	353	20–59	Luzerna	Aneroid, BPLM & SRH	140/90
Rosário TM	LILACS, Scielo & CAPES-TD	2006	1003	18–90	Nobres	Oscillometric	140/90
Braga Junior FD	CAPES-TD	2007	1298	20–59	Cuiabá	Device not described	140/90
Chrestani MAD	LILACS, Scielo & CAPES-TD	2007	2910	≥20	Pelotas	Wrist Oscillometric, BPLM & SRH	140/90
Longo GZ	Scielo	2007	2022	20–59	Lages	Oscillometric & BPLM	140/90

**Table 2:** Meta-analysis of observational studies: prevalence rate of hypertension by decade and adjustment to the design effect, including 12 2018 individuals.

Decade	Hypertension criteria (number of studies/number of adjusted studies)	Prevalence rate (95% CI)				Adjusted *vs.* unadjusted*
		Males	Females	Overall (all studies)	Overall (adjusted studies)	
1980’s	WHO (n = 5/1)	21.6 (14.9–30.2)	18.0 (11.3–27.4)	23.6 (17.3–31.4)	31.3 (28.6–34.2)	<0.001
	JNC (n = 3/2)	45.1 (40.0–50.4)	34.6 (23.7–47.5)	36.1 (28.7–44.2)	36.7 (24.4–51.0)	0.57
1990’s	WHO (n = 6/0)	20.3 (17.0–24.1)	20.02 (14.4–27.6)	19.6 (16.4–23.3)	–	–
	JNC (n = 8/0)	29.7 (22.5–38.2)	27.2 (19.9–36.1)	32.9 (29.9–36.0)	–	–
2000’s	Self-report in home visit (n = 4/2)	15.8 (11.7–21.0)	23.4 (16.6–31.9)	25.2 (23.3–27.2)	20.0 (14.4–27.1)	<0.001
	Self-report through telephone inquiry (n = 5/4)	18.6 (17.4–19.9)	23.2 (21.1–25.4)	20.6 (19.0–22.4)	21.4 (20.3–22.6)	0.51
	JNC (n = 14/4)	27.3 (22.5–32.8)	27.7 (23.7–32.0)	28.7 (26.2–31.4)	30.7 (26.6–35.1)	0.07

Decade	Hypertension criteria (number of studies)	χ^2^ (P value)					
1980’s *vs.* 1990’s	WHO (n = 11)	0.33	0.10	<0.001			
1980’s *vs.* 1990’s	JNC (n = 11)	<0.001	<0.001	0.02			
1980’s *vs.* 2000’s	JNC (n = 17)	<0.001	0.006	<0.001			
1980’s to 2000’s	JNC (n = 25)	<0.001**	<0.001**	<0.001**			

WHO = World Health Organization diagnostic criteria; JNC = Joint National Committee diagnostic criteria; 95% CI = 95% confidence interval.

* P value for χ^2^.

** P value for χ for trend.

Prevalence of hypertension by the former WHO criteria in older studies was obviously lower than the prevalence by the JNC criteria. Self-reported hypertension, either at home or by telephone interview (mostly previous doctor’s diagnoses), yielded lower prevalence rates as well. Prevalence rates were roughly similar among men and women and did not change substantially in studies with adjustment for the design effect or using different blood pressure devices. Heterogeneity was present in all the pooled estimates shown in Table 2 (P<0.001 and *I^2^*>90.0% for every analyses).

**Figure 2:**
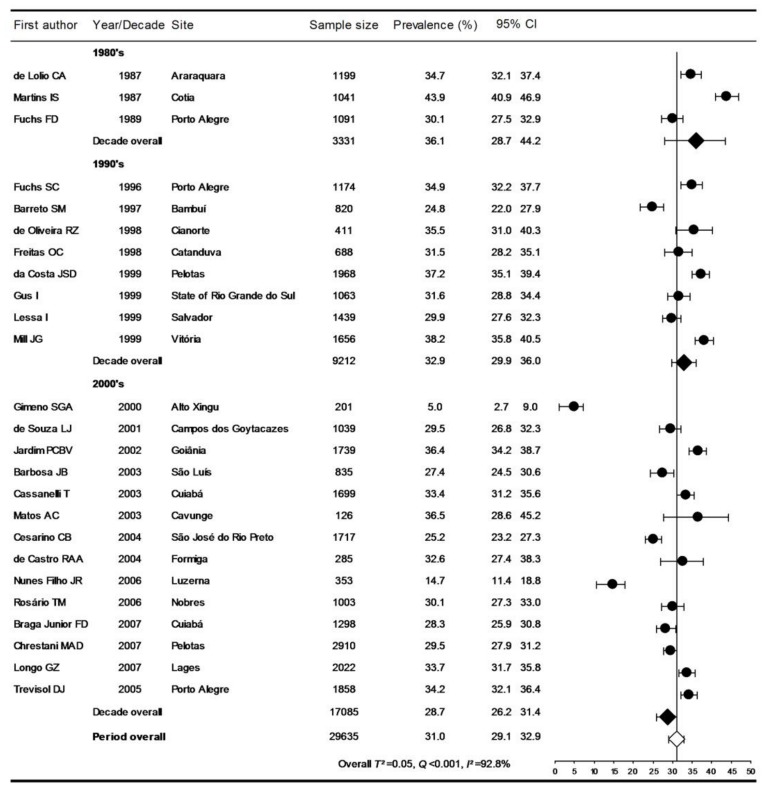
Prevalence of hypertension, according to the JNC criteria, by decade.

**Figure 3:**
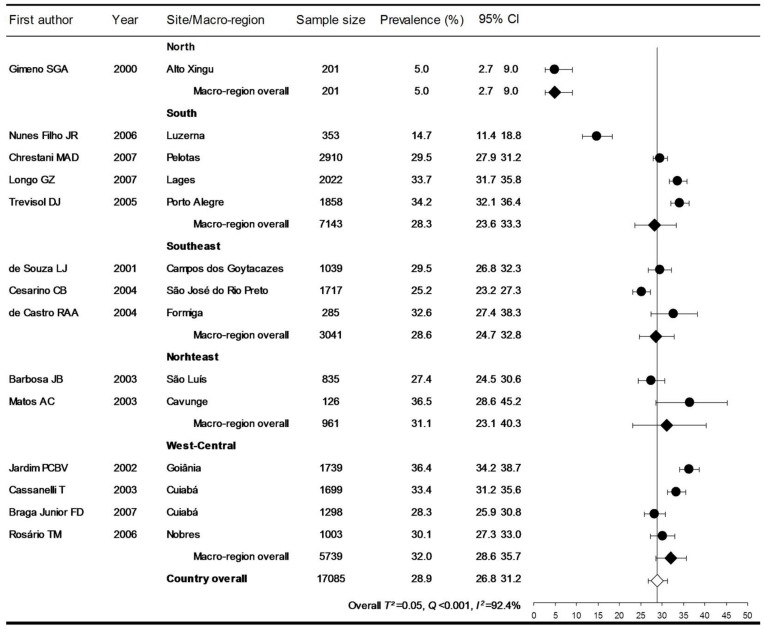
Prevalence of hypertension, according to the JNC criteria, by Brazilian macro-region in the 2000’s.

Prevalence rates according to the JNC criteria in individual studies, summary estimates by decade, and overall pooled rate are presented in Figure 2. The prevalence decreased by decades: 36.1% (28.7–44.2) in the 1980’s, 32.9% (29.9–36.0) in the 1990’s, and 28.7 (26.2–31.4) in the 2000’s (P for trend <0.001). The estimated prevalence for the past three decades (according to the JNC criteria) was 31.0%, with 95% CI from 29.1 to 32.9%. With the exception of the North macro-region, which had estimates of prevalence exclusively from the Alto Xingu Indian population, the prevalence was similar among the various Brazilian macro-regions (Figure 3).

**Figure 4:**
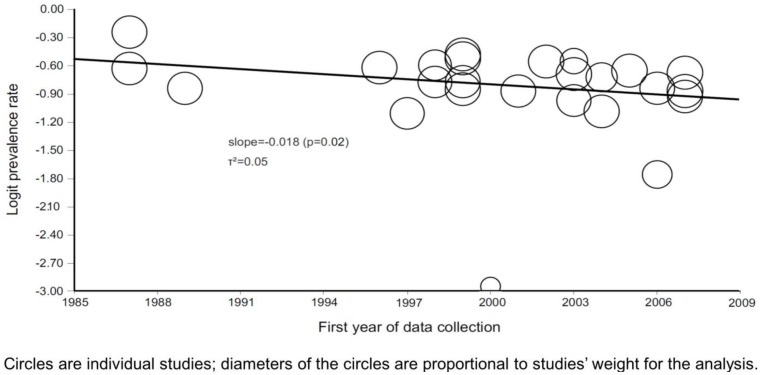
Regression of first year of data collection on logit prevalence rate according to the JNC criteria.

In the 2000’s, pooled prevalence rate for studies adjusted for the design effect did not differ from all studies (adjusted and unadjusted) according to the JNC criteria (χ^2^ P = 0.07) and telephone inquiries (P = 0.51). The meta-regression of year of data collection over logit prevalence confirmed a trend toward decreasing in prevalence from 1987 to 2007, with a slope of −0.018 (P = 0.02). Furthermore, a τ^2^ = 0.05 was found, which means that differences in the year of data collection explain 90.2% of the between-studies variance (Figure 4).

Meta-regression of year of data collection over logit prevalence according to sex showed a non-significant slope of −0.012 (P = 0.42) for women, and a significant slope of −0.035 (P = 0.02) for men (τ^2^ = 0.11; explained between-studies variance of 79.2%) (data not shown). Meta-regression of HDI on logit prevalence (according to 2000 HDI for each city) retrieved a non-significant slope of 1.070 (P = 0.42) (data not shown). Additionally, control rates were properly reported in 10 studies and pooled rates, according to the JNC criteria, were 33.8% (26.0–42.6%), 28.1% (23.7–32.7%), and 24.1% (10.1–47.3%) in the 1980’s, 1990’s and the 2000’s, respectively (χ^2^ for trend p<0.001).

### Assessing Bias

**Figure 5:**
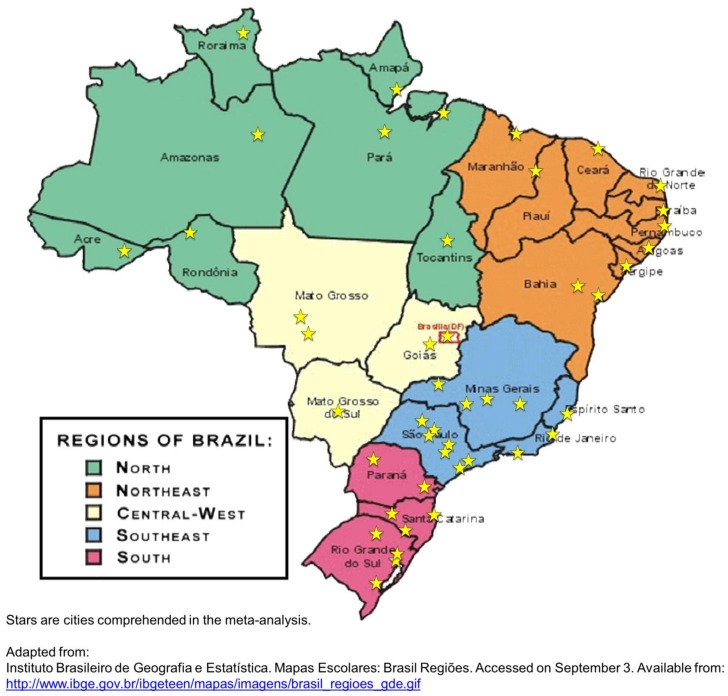
Map of Brazil according to its five macro-regions with the cities comprehended in the meta-analysis.

All studies were cross-sectional, and there was moderate (59.0%) overlap of records across different databases. Five studies (12.5%) were from the 1980’s, 11 (27.5%) from the 1990’s and 24 (57.5% to 60.0%) from 2000’s. Sample sizes varied substantially with a median of 1268 (IQR 838.5). Most studies that measured blood pressure employed aneroid or mercury manometers (18 studies), and eight used oscillometric manometers. Almost all studies were from urban populations (37 studies), and mostly were done in the South and Southeast macro-regions of Brazil (Figure 5). In regard to methodological features of the studies, 33 used multistage cluster sampling, six used simple random sampling, and the study by Gimeno et al. evaluated 90% of the adults of Alto Xingu’s native Brazilian ]10]. Most studies (n = 25; 62.5%) did not have selection bias with potential to compromise their internal validity. Fourteen (35.0%) studies had sampling or sample size calculation poorly described. Only one study had high rate of missing data. In 10 studies the first measurement was discarded. Twelve (30.0%) studies, mostly done in the 2000’s, presented data adjusted for design effect. Table S1 presents data on potential selection and measurement biases, as well as bias in the analysis.

Sensitivity analyses were carried out excluding studies conducted in rural areas (n = 3), studies that did not investigate elderly individuals (n = 2), studies that employed oscillometric wrist manometer (n = 1), and one with a small sample size [11]–[16]. The overall prevalence for the decade did not altered significantly (30.8%; 95%CI: 27.8–34.0%). All other sensitivity analyses defined *a priori* (see Assessment of study quality and risk of bias) showed similar results with no statistically significant differences (data not shown).

## Discussion

In this comprehensive systematic review with meta-analysis of cross-sectional surveys done in Brazil in the last three decades, including more than 120 thousand individuals, it was possible to compute precise estimates of prevalence by decade, by criteria of definition of hypertension, by methods of diagnosing hypertension, and by gender. Overall, the prevalence was similar to described in developed countries, particularly of hypertension diagnosed by blood pressure measurement and based on the contemporaneous universal criteria for diagnosis of hypertension, and without any substantial differences by gender [1], [17]. An apparent trend to lowering in prevalence by decade was evident. The proportion of one-third of hypertensive individuals with controlled blood pressure is also within the range of rates of control described worldwide [18].

Our study could circumvent many limitations of individuals studies selected for the meta-analysis of Danaei et al, such as regional inequities [19]. Furthermore, Danaei et al employed mean systolic blood pressure to describe trends of risk, an approach that does not take into account the real number of subjects at risk. The potential reasons for bias in the whole estimates are the overrepresentation of studies done in metropolitan populations, particularly from the South and Southeast macro-regions of the country. Nonetheless, 84.4% of the Brazilian population lives nowadays in cities [20]. The absence of representative data from the North macro-region was partially overcome by the inclusion of a study of native Brazilians. On the other hand, the North macro-region, although has the largest area, has the lowest density in the country, comprising 50% of Brazil’s land territory, but only 5% of the country’s population [20]. A few studies enrolled subjects below the age range, but the analysis with and without those studies did not change substantially the overall estimates.

Prevalence rates based on direct measurement of blood pressure were higher than those based on self-report hypertension [21]–[23]. The lower prevalence in telephone surveys may additionally be secondary to the differential distribution of telephones by social classes, leading to an underrepresentation of individuals from lower classes, who had higher prevalence of hypertension [22], [24]–[25].

Most studies did not take into account the distortions caused by multistage and weighting sampling. The lack of adjustment for design effect can compromise accuracy of prevalence confidence intervals for individual studies and, consequently, making the results of older surveys less reliable than those done in the last decade [26], [27]. Nevertheless, the comparison between studies with and without adjustment for sampling design showed that the former provided reliable estimates.

The average absolute reduction in prevalence of 3.7% per decade is consistent with recent meta-analysis that found a mean 1.8 and 3.5 mmHg decrease per decade in systolic blood pressure for males, and females, respectively, from 1980 to 2008 [7]. Also, meta-regression showed a slight, but steady relative reduction in prevalence of 1.8% per year from 1987 to 2007. This trend reproduces the estimates observed in industrialized nations, confirming that the epidemiological transition already finished in Brazil in regard to hypertension. Significant reduction in prevalence among men and non-significant reduction in women might suggest that the overall prevalence decrease had a greater impact in men.

The trend toward reduction of the control rate was contrary to expectations. Increase in detection of hypertension and of the access to BPLM in the Brazilian Health System (universal coverage and free of charge), in the 1990’s. Hence, the number of subjects on treatment for hypertension might have augmented proportionally more than the number of subjects with controlled hypertension in the last two decades. It might give the false impression that fewer subjects are keeping their blood pressure below 140/90 mm Hg. Nonetheless, the pooled estimate of control rate is consistent with the literature [28].

Despite the heterogeneity of studies, lack of adjustment for effect design in many studies, and underrepresentation of the population from the North macro-region, the estimates are reliable and within the range of prevalence described for industrialized nations. The trend for lowering in the prevalence rates by decade follows the pattern of industrialized countries as well. The proportion of individuals with controlled hypertension, of about one-third of individuals, is similar to the described in other countries, and it requires innovative and effective means to improve the rates of control.

This pooled analysis of prevalence of hypertension is an attempt to fill the lack of national data. However, the estimates of prevalence of hypertension not adequately represent the Brazilian Indians, the rural population, and those living in the vicinity of the Amazon rainforest. This study presents data for the most populated areas of Brazil, as can be seen in the Brazil map (Figure 5). Therefore, the results are not a substitute for a nationwide prevalence study. Therefore, the results are not a substitute for a national prevalence study. However, until this study be conducted, these analyzes are the best estimates available that can serve as a reference for public health policy [29].

### Conclusions

As such, this meta-analysis was an alternative way to establishing the hypertension prevalence in Brazil, which is necessary to assess the hypertension burden and to implement cost-effective interventions. Nonetheless, a nationwide prevalence study is still needed to confirm the estimates and determine more accurate rates for specific populations.

## Supporting Information

Table S1 **List of studies selected for the systematic review and the reasons for exclusion of studies.** The table has all potentially eligible studies which were not included in the systematic review.
(DOC)

Table S2 **Spreadsheet for extraction of data of studies selected for the systematic review.** The spreadsheet may be useful to those who are going to conduct a systematic review.
(XLS)

Table S3 **Review protocol of the Systematic Review.**
(DOC)

Table S4 **PRISMA 2009 Checklist.**
(DOC)

## References

[1] Kearney PM, Whelton M, Reynolds K, Muntner P, Whelton PK, et al (2005) Global burden of hypertension: analysis of worldwide data. Lancet 365: 217–223. 15652604 10.1016/S0140-6736(05)17741-1

[2] Mittal BV, Singh AK (2010) Hypertension in the developing world: challenges and opportunities. Am J Kidney Dis 55: 590–598. 19962803 10.1053/j.ajkd.2009.06.044

[3] Lewington S, Clarke R, Qizilbash N, Peto R, Collins R, et al (2002) Age-specific relevance of usual blood pressure to vascular mortality: a meta-analysis of individual data for one million adults in 61 prospective studies. Lancet 360: 1903–1913. 12493255 10.1016/s0140-6736(02)11911-8

[4] von Elm E, Altman DG, Egger M, Pocock SJ, Gøtzsche PC, et al (2007) The Strengthening the Reporting of Observational Studies in Epidemiology (STROBE) statement: guidelines for reporting observational studies. Epidemiology 18: 800–804. 18049194 10.1097/EDE.0b013e3181577654

[5] Stroup DF, Berlin JA, Morton SC, Olkin I, Williamson GD, et al (2000) Meta-analysis of observational studies in epidemiology: a proposal for reporting. Meta-analysis Of Observational Studies in Epidemiology (MOOSE) group. JAMA 283: 2008–12. 10789670 10.1001/jama.283.15.2008

[6] Chobanian AV, Bakris GL, Black HR, Cushman WC, Green LA, et al (2003) The Seventh Report of the Joint National Committee on Prevention, Detection, Evaluation, and Treatment of High Blood Pressure: the JNC 7 report. JAMA 289: 2560–2572. 12748199 10.1001/jama.289.19.2560

[7] Report of an Expert Committee. Arterial Hypertension and Ischaemic Heart Disease: Preventive Aspects. Available: http://whqlibdoc.who.int/trs/WHO_TRS_231.pdf. Accessed 2011 Aug 10.

[8] Neyeloff JL, Fuchs SC, Moreira LB (2012) Meta-analyses and Forest Plots using a Microsoft Excel spreadsheet: step-by-step guide focusing on descriptive data analysis. BMC Research Notes 5: 52. 22264277 10.1186/1756-0500-5-52 PMC3296675

[9] Trevisol DJ, Moreira LB, Fuchs FD, Fuchs SC (2012) Health-related quality of life is worse in individuals with hypertension under drug treatment: results of population-based study J Hum Hypertens. 26: 374–380. 10.1038/jhh.2011.48 21593782

[10] Gimeno SGA, Rodrigues D, Pagliaro H, Cano EN, de Souza Lima EE, et al (2007) Metabolic and anthropometric profile of Aruák Indians: Mehináku, Waurá and Yawalapití in the Upper Xingu, Central Brazil, 2000–2002. Cad Saude Publica 23: 1946–1954. 17653412 10.1590/s0102-311x2007000800021

[11] Longo GZ, Neves J, Luciano VM, Peres MA (2009) Prevalence of high blood pressure levels and associated factors among adults in Southern Brazil. Arq Bras Cardiol 93: 387–394. 19936459 10.1590/s0066-782x2009001000012

[12] Braga Jr F (2009) [Hypertension and Physical Activity in Cuiabá: Population Based Study] [Masters Dissertation]. Cuiabá, MT: Universidade Federal do Mato Grosso; 2007.

[13] Matos AC, Ladeia AM (2003) Assessment of cardiovascular risk factors in a rural community in the Brazilian state of Bahia. Arq Bras Cardiol 81: 291–302. 14569373 10.1590/s0066-782x2003001100009

[14] Nunes FilhoJR, Debastiani D, Nunes AD, Peres KG (2007) Prevalence of cardiovascular risk factors in adults living in Luzerna, Santa Catarina, in 2006. Arq Bras Cardiol 89: 289–293. 18066452 10.1590/s0066-782x2007001700007

[15] Chrestani MA, Santos Ida S, Matijasevich AM (2009) Self-reported hypertension: validation in a representative cross-sectional survey. Cad Saude Publica 25: 2395–2406. 19936478 10.1590/s0102-311x2009001100010

[16] Castro RA, Moncau JE, Marcopito LF (2007) Hypertension prevalence in the city of Formiga, MG, Brazil. Arq Bras Cardiol 88: 334–339. 17533476 10.1590/s0066-782x2007000300013

[17] Kearney PM, Whelton M, Reynolds K, Whelton PK, He J (2004) Worldwide prevalence of hypertension: a systematic review. J Hypertens 22: 11–19. 15106785 10.1097/00004872-200401000-00003

[18] Whelton PK, He J, Muntner P (2004) Prevalence, awareness, treatment and control of hypertension in North America, North Africa and Asia. J Hum Hypertens 18: 545–551. 15269704 10.1038/sj.jhh.1001701

[19] Danaei G, Finucane MM, Lin JK, Singh GM, Paciorek CJ, et al.; Global Burden of Metabolic Risk Factors of Chronic Diseases Collaborating Group (Blood Pressure) (2011) National, regional, and global trends in systolic blood pressure since 1980: systematic analysis of health examination surveys and epidemiological studies with 786 country-years and 5·4 million participants. Lancet 377: 568–577. 21295844 10.1016/S0140-6736(10)62036-3

[20] Instituto Brasileiro de Geografia e Estatística. Sinopse do Censo Demográfico 2010. Available: http://www.ibge.gov.br/home/estatistica/populacao/censo2010/sinopse.pdf. Accessed 2011 May 30.

[21] Bowlin SJ, Morrill BD, Nafziger AN, Jenkins PL, Lewis C, et al (1993) Validity of cardiovascular disease risk factors assessed by telephone survey: the Behavioral Risk Factor Survey. J Clin Epidemiol 46: 561–571. 8501483 10.1016/0895-4356(93)90129-o

[22] Bowlin SJ, Morrill BD, Nafziger AN, Lewis C, Pearson TA (1996) Reliability and changes in validity of self-reported cardiovascular disease risk factors using dual response: the behavioral risk factor survey. J Clin Epidemio 49: 511–507. 10.1016/0895-4356(96)00010-8 8636724

[23] Giles WH, Croft JB, Keenan NL, Lane MJ, Wheeler FC (1995) The validity of self-reported hypertension and correlates of hypertension awareness among blacks and whites within the stroke belt. Am J Prev Med 11: 163–169. 7662395

[24] Segri NJ, Cesar CL, Barros MB, Alves MC, Carandina L, et al (2010) Health survey: comparison of interviewees according to ownership of a residential telephone line. Rev Saude Publica 44: 503–512. 20464258 10.1590/s0034-89102010005000012

[25] Fuchs FD, Moreira LB, Moraes RS, Bredemeier M, Cardozo SC (1994) Prevalence of systemic arterial hypertension and associated risk factors in the Porto Alegre metropolitan area. Populational-based study. Arq Bras Cardiol 63: 473–479. 7605231

[26] Guillén M, Ayuso M (2004) The importance of the sample design effect. Med Clin (Barc) 122: 35–38. 14980158 10.1157/13057544

[27] Office for National Statistics. Health Statistics Quarterly 45 Spring 2010. Available: http://www.statistics.gov.uk/hsq/downloads/HSQ45.pdf. Accessed 2011 Sep 7.

[28] Pereira M, Lunet N, Azevedo A, Barros H (2009) Differences in prevalence, awareness, treatment and control of hypertension between developing and developed countries. J Hypertens 27: 963–975. 19402221 10.1097/hjh.0b013e3283282f65

[29] Tomson J, Lip GY (2005) Blood pressure demographics: nature or nurture … … genes or environment? BMC Med 3: 3. 15638936 10.1186/1741-7015-3-3 PMC544878

