# Supplementary material for: Trends in Prevalence of Hypertension in Brazil: A Systematic Review with Meta-Analysis
Source: PLoS One. 2012 Oct 31;7(10):e48255. doi: 10.1371/journal.pone.0048255 (PMC3485225; doi:10.1371/journal.pone.0048255)
Supplement: Table S1 — List of studies selected for the systematic review and the reasons for exclusion of studies. The table has all potentially eligible studies which were not included in the systematic review. (DOC) [file pone.0048255.s001.doc]

| Table S1. List of studies selected for the systematic review and the reasons for exclusion of studies | | |
| --- | --- | --- |
| **First Author** | **Year of publication** | **Eligibility** |
| Achutti A | 1994 | Excluded - Data prior to 1980 |
| Ala L | 2004 | Excluded - Not population-based: restricted to certain socioeconomic stratum |
| Alves LM | 2007 | Excluded - Not population-based: restricted to outpatient clinics |
| Ayres JE | 1991 | Included |
| Ayres JE | 1990 | Excluded - Review article |
| Barbieri MA | 2006 | Excluded - Other definition of hypertension |
| Barbosa JB | 2008 | Included |
| Barbosa PJB | 2006 | Excluded - Duplicate data |
| Barreto ML | 1980 | Excluded - Data prior to 1980 |
| Barreto SM | 2001 | Included |
| Barreto SM | 2003 | Excluded - Duplicate data |
| Barros FC | 1999 | Excluded - No data on adults |
| Barros MBA | 2006 | Excluded - Review article |
| Bloch KV | 1994 | Included |
| Bloch KV | 2003 | Excluded - Duplicate data |
| Borges HP | 2008 | Included |
| Braga Junior FD | 2007 | Included |
| Bulbol WS | 1981 | Excluded - Other definition of hypertension |
| Bustos P | 2007 | Excluded - Duplicate data |
| Cabral PC | 1983 | Excluded - Not population-based: restricted to outpatient clinics |
| Capilheira MF | 2004 | Excluded - No report on hypertension prevalence |
| Capilheira MF | 2008 | Included |
| Caranti DA | 2008 | Excluded - No data on adults |
| Cardoso AM | 2001 | Excluded - Sampling inadequately described |
| Carneiro G | 2003 | Excluded - Not population-based: prevalence among the obese |
| Carvalhaes MABL | 2008 | Included |
| Carvalho JJ | 1983 | Excluded - Data prior to 1980 |
| Cassanelli T | 2005 | Included |
| Cercato C | 2004 | Excluded - Not population-based: restricted to outpatient clinics |
| Cesarino CB | 2008 | Included |
| Chrestani MAD | 2009 | Included |
| Coimbra Jr CE | 2001 | Excluded - Full-article unavailable |
| Cordeiro | 1998 | Excluded - Not population-based: restricted to certain working class |
| Costa EA | 1990 | Excluded - Data prior to 1980 |
| Costa VG | 1984 | Included |
| Curzio EMFO | 2003 | Excluded - Not population-based: restricted to outpatient clinics |
| da Costa AS | 1986 | Excluded - Other definition of hypertension |
| da Costa JSD | 2007 | Excluded - Duplicate data |
| da Costa JSD | 2002 | Included |
| de Aquino EMMLL | 2001 | Excluded - Not population-based: restricted to certain working class |
| de Castro RAA | 2007 | Included |
| de Lólio CA | 1989 | Excluded - Thesis/dissertation whose article was included |
| de Lolio CA | 1990 | Included |
| de Lolio CA | 1990 | Excluded - Review article |
| de Lolio CA | 1993 | Excluded - Duplicate data |
| de Oliveira NMCM | 2005 | Excluded - Not population-based: restricted to certain socioeconomic stratum |
| de Oliveira RZ | 2003 | Included |
| de Sousa LB | 2006 | Excluded - Not population-based: restricted to outpatient clinics |
| de Souza ARA | 2007 | Excluded - Not population-based: non-probabilistic sampling |
| de Souza JJG | 2006 | Included |
| de Souza LJ | 2003 | Included |
| Dressler WW | 1991 | Excluded - Full-article unavailable |
| Duda NT | 1994 | Excluded - Review article |
| Duncan BB | 1993 | Excluded - Not population-based: non-probabilistic sampling |
| Dutra CLC | 2004 | Excluded - No data on adults |
| Feijão AM | 2005 | Excluded - Not population-based: restricted to certain socioeconomic stratum |
| Ferreira SRG | 2009 | Included |
| Florencio TT | 2004 | Excluded - Not population-based: restricted to certain socioeconomic stratum |
| Formigli VLA | 1998 | Excluded - Not population-based: non-probabilistic sampling |
| Franco GPP | 2009 | Excluded - No report on hypertension prevalence |
| Freitas OC | 2001 | Included |
| Fuchs FD | 1997 | Excluded - Not population-based: restricted to outpatient clinics |
| Fuchs FD | 1994 | Excluded - Review article |
| Fuchs FD | 1995 | Excluded - Not population-based: restricted to outpatient clinics |
| Fuchs FD | 1994 | Included |
| Fuchs SC | 2008 | Included |
| Fuchs SC | 2001 | Included |
| Fuzikawa AK | 2008 | Excluded - No data on adults |
| Gigante DP | 2009 | Excluded - Duplicate data |
| Gimeno SGA | 2007 | Included |
| Gomes BMR | 2007 | Excluded - Not population-based: data restricted to students |
| Guimarães AC | 2002 | Excluded - Review article |
| Gus I | 2002 | Included |
| Gus I | 2004 | Excluded - Duplicate data |
| Gus M | 2004 | Excluded - Duplicate data |
| Gus M | 1998 | Excluded - Duplicate data |
| Hartmann M | 2007 | Included |
| Hasselmann MH | 2008 | Excluded - Not population-based: restricted to certain working class |
| IBGE* | 2003 | Excluded - No report on hypertension prevalence |
| INCA** | 2004 | Included |
| James SA | 1991 | Excluded - Review article |
| Jardim PCBV | 2007 | Included |
| Klein CH | 1985 | Excluded - Data prior to 1980 |
| Klein CH | 1995 | Excluded - No report on hypertension prevalence |
| Klein CH | 1995 | Excluded - Duplicate data |
| Lessa I | 1981 | Excluded - Not population-based: data restricted to students |
| Lessa I | 2004 | Excluded - No report on hypertension prevalence |
| Lessa I | 2006 | Included |
| Lima-Costa MF | 2004 | Excluded - Duplicate data |
| Longo GZ | 2009 | Included |
| Lubianca Neto JF | 1997 | Excluded - Not population-based: restricted to outpatient clinics |
| Magalhães MOC | 2008 | Excluded - No data on adults |
| Makdisse M | 2008 | Excluded - Definition of hypertension not reported |
| Manfroi WC | 2002 | Excluded - Not population-based: patients with acute myocardial infarction |
| Marcopito LF | 2005 | Excluded - Sampling inadequately described |
| Marquezine GF | 2008 | Excluded - Duplicate data |
| Martins IS | 1997 | Included |
| Martins IS | 1989 | Excluded - No report on hypertension prevalence |
| Masson CR | 2004 | Excluded - Thesis/dissertation whose article was included |
| Matos AC | 2003 | Included |
| Mesquita CMB | 2008 | Excluded - Thesis/dissertation whose article was included |
| Mill JG | 2004 | Included |
| Molina MCB | 2003 | Excluded - Duplicate data |
| Monteiro CA | 2005 | Included |
| Moreira LB | 1998 | Excluded - Duplicate data |
| Muccini AR | 1993 | Excluded - Not population-based: subjects accessed in a Health Fair of the city |
| Nakazone MA | 2007 | Excluded - Not population-based: patients with hypertension and CVD risk factors of a private clinic |
| Neves EB | 2008 | Excluded - Not population-based: male members of the Brazilian Army Post-Graduation School for Officers |
| Nissinen A | 1988 | Excluded - Review article |
| Nunes Filho JR | 2007 | Included |
| Olinto MTA | 2004 | Excluded - Duplicate data |
| Oliveira EP | 2006 | Excluded - Other definition of hypertension |
| Passos VMA | 2006 | Excluded - Review article |
| Peixoto MRG | 2008 | Included |
| Pereira JC | 2009 | Excluded - Excluded - Duplicate data |
| Pereira MR | 2007 | Excluded - Not population-based: subject selected from a list of electric power connections |
| Pereira RA | 1999 | Excluded - No report on hypertension prevalence |
| Piccini RX | 1994 | Included |
| Pimenta AM | 2005 | Excluded - Thesis/dissertation whose article was included |
| Pimenta AM | 2008 | Excluded - Duplicate data |
| Polidoro AA | 2008 | Excluded - Not population-based: non-probabilistic sampling of undergraduate students from Maringá University |
| Pousada JMDC | 2006 | Excluded - Not population-based: Spaniards and their descendants presently living in Salvador and registered with the Spanish Consulate or at the Spanish Hospital as members of Spanish community in Salvador, Brazil |
| Rego RA | 1990 | Excluded - Not population-based: non-probabilistic sampling of 8 sectors of the city, being two of low socioeconomic status |
| Reichert FF | 2009 | Excluded - Duplicate data |
| Ribeiro AB | 1986 | Excluded - Review article |
| Ribeiro MD | 1982 | Excluded - Not population-based: workers from labor force of the Metropolitan region of São Paulo |
| Ribeiro RQC | 2003 | Excluded - Not population-based: school-based sampling of subjects aged 6 to 18 years. |
| Rodrigues SL | 2006 | Excluded - Duplicate data |
| Rosário TM | 2009 | Included |
| Rosenbaum P | 2005 | Excluded - Not population-based: Japanese-  Brazilian population over 30 years of age invited to participate. |
| Rosini N | 2006 | Excluded - Not population-based: a population sample of hypertensive smokers diagnosed and enrolled at the *Hiperdia* Program of the Ministry of Health |
| Sabry MOD | 2002 | Excluded - Not population-based: employees of a university in the city of Fortaleza, in state of Ceará, Fortaleza, Brazil. |
| Salaroli LB | 2007 | Excluded - No report on hypertension prevalence |
| Sarno F | 2007 | Excluded - Not population-based: a sample of employees from a private general hospital |
| Sawaya AL | 2005 | Excluded - Review article |
| Schmidt MI | 2009 | Excluded - Duplicate data |
| Schwingel A | 2007 | Excluded - Not population-based: subjects from an urban areas in Japan and Brazil |
| Sichieri R | 2001 | Excluded - Full-article unavailable |
| Sichieri R | 2000 | Excluded - refusal to participate ≥25% |
| Silva GEC | 2004 | Excluded - Not population-based: |
| Silva MAD | 1998 | Excluded - Not population-based: patients were selected in 20 medical centers in Brazil |
| Simony RF | 2007 | Excluded - Not population-based: subjects from the first and second-generation Japanese-brazilians |
| Siqueira AFA | 2007 | Excluded - Not population-based: non-mixed population of subjects from the first and second-generation Japanese-brazilians living in Bauru |
| SOFT*** | 2007 | Included |
| Sparrenberger | 2008 | Excluded – Duplicate data |
| Stamm AMNF | 2007 | Excluded - Not population-based: hypertensive patients undergoing treatment at the Internal Medicine and Cardiology Outpatient Clinics at a University Hospital in the Southern Region of Brazil. |
| Teichmann LM | 2005 | Excluded - No report on hypertension prevalence |
| Teodósio MR | 2004 | Excluded - Not population-based: mothers of students enrolled at schools of Jaboatão dos Guararapes, Pernambuco, Brasil |
| Trindade IS | 1998 | Included |
| van Eyken EBBDO | 2009 | Excluded - Not population-based: men 20 to 49 years of age, from a list of residents within this age range of Family Medicine Service |
| Velasquez-Melendez G | 2007 | Excluded - Not population-based: five settlements, each located between 1 and 5 km from Virgem das Graças, a rural village in the municipality of Ponto dos Volantes, situated in a semiarid region of the Jequitinhonha Valley in the state of Minas Gerais, Brazil. It was a convenient sampling. |
| Velásquez-Meléndez G | 2002 | Excluded - Not population-based: Participants were female volunteers in apparent good general health, with no chronic or acute metabolic or infectious complaints, who were treated at the various departments of the Health Center, with the objective of obtaining a wide range of age and BMI values. |
| Wiehe M | 2004 | Excluded - Thesis/dissertation whose article was included |
| Wiehe M | 2006 | Excluded - Duplicate data |
| Yunis C | 1998 | Excluded - Review article |
| Zaitune MPA | 2006 | Excluded - No data on adults |
| * The Brazilian Institute for Geography and Statistics  ** The Brazilian National Cancer Institute  *** The Syndrome of Obesity and Cardiovascular Risk Factors Study | | |
