# Supplementary material for: Trends in Prevalence of Hypertension in Brazil: A Systematic Review with Meta-Analysis
Source: PLoS One. 2012 Oct 31;7(10):e48255. doi: 10.1371/journal.pone.0048255 (PMC3485225; doi:10.1371/journal.pone.0048255)
Supplement: Table S3 — Review protocol of the Systematic Review. (DOC) [file pone.0048255.s003.doc]

**Review protocol of the Systematic Review:**

**1.Search strategy:**

a) Key words: “hypertension”, “prevalence”, “statistics”, and “Brazil”

b) PubMed search: ("Hypertension"[Majr] AND "Prevalence") AND "Brazil" limited to all adults (≥19 years-old), and ("Hypertension/epidemiology"[Majr] OR "Hypertension/statistics and numerical data"[Majr]) AND "Brazil" limited to all adults (≥18 years-old).

c) Embase search: 'hypertension'/exp/mj AND 'prevalence'/de AND 'brazil'/exp AND ([article]/lim OR [article in press]/lim) AND [adult]/lim AND [humans]/lim

d) Screened: titles and abstracts

e) Excluded: secondary hypertension and non-probabilistic sampling*

* A probability sampling should describe: eligible population, sampling procedure, studied population (refusal or loose rate)

f) Assessed: full-text reading

g) Compared the lists of selected papers by the reviewers.

h) Disagreement among reviewers: called a third reviewer and decision for inclusion reached by consensus.

**2.Data extraction:**

a) Spreadsheet designed based on the Strengthening the Reporting of Observational Studies in Epidemiology Statement (STROBE) checklist: items 4, 5, 6a, 7–10, 12c–e, 13a, 14b, 16a, and 17 (see supplementary file).
